# Diverse Manifestations of COVID-19: Some Suggested Mechanisms

**DOI:** 10.3390/ijerph18189785

**Published:** 2021-09-17

**Authors:** Md S. Zaman, Robert C. Sizemore

**Affiliations:** 1 Department of Biological Sciences, Alcorn State University, Lorman, MS 39096, USA; sizemore@alcorn.edu; 2 Department of Biology, South Texas College, McAllen, TX 78501, USA

**Keywords:** SARS-CoV-2, COVID-19, coronaviruses, clinical manifestations, BCG, MMR vaccines, angiotensin I and II, ACE2 receptors, asymptomatic infection

## Abstract

Severe acute respiratory syndrome coronavirus 2 (SARS-CoV-2), the cause of the novel respiratory disease COVID-19, has reached pandemic status and presents a wide range of manifestations of diverse magnitude, including fever, cough, shortness of breath, and damage to vital organs, such as the heart, lung, kidney, and brain. Normally, older individuals and those with underlying health issues are more at risk. However, about 40% of COVID-19 positive individuals are asymptomatic. This review aims to identify suggested mechanisms of diverse manifestations of COVID-19. Studies suggest that T cell-mediated immunity and specific and/or nonspecific immunity from other vaccines could protect against SARS-CoV-2. The potential role of cross-reacting antibodies to coronaviruses that cause the common cold, mumps virus, polio virus, and pneumococcal bacteria are also suggested to help protect against COVID-19. Decreased production of Type I interferons (IFN-α and IFN-β) could also be linked to COVID-19 manifestations. Several studies suggest that ACE2 cell membrane receptors are involved in SARS-CoV-2 infection. However, the relationship between an abundance of ACE2 receptors and the infectivity of the virus is unknown. Unlocking these manifestation mysteries could be crucial as this could help researchers better understand the virulence, pathology, and immune responses associated with SARS-CoV-2, leading to the development of effective therapies and treatment plans.

## 1. Introduction

Coronaviruses are a large family of viruses that are common in humans and several animals, such as cattle, camels, cats, and bats. Commonly known human coronaviruses that cause common colds and upper respiratory infections are HCoV-229E (α-coronavirus), HCoV-NL63 (α-coronavirus), HCoV-OC43 (β-coronavirus), and HCoV-HKU1 (β-coronavirus). However, they can also contribute to life-threatening lower respiratory infections in infants, the elderly, and immunocompromised patients [1]. Coronaviruses that infect animals can also evolve and cause human infections. Common examples of such cases are two earlier viruses, SARS-CoV (Severe Acute Respiratory Syndrome) and MERS-CoV (Middle East Respiratory Syndrome), followed by the current virus SARS-CoV-2. All three of these viruses are thought to have originated in bats and eventually infected humans. However, the exact source of SARS-CoV-2 is yet to be confirmed.

SARS-CoV-2 was first identified in an outbreak of respiratory infection in Wuhan, China, in December 2019. The infectious respiratory disease caused by this virus is now known as Coronavirus Disease 2019 (*COVID-*19**). The virus spread very quickly from China and infected 221 countries, reaching pandemic status [2]. As of this writing, according to the Center for Systems Science and Engineering (CSSE) of Johns Hopkins University real-time data tracking, SARS-CoV-2 has infected over 219.4 million people and caused 4.5 million deaths worldwide. In the United States alone, the virus has infected 39.6 million people and inflicted over 643,000 deaths [3]. The Institute for Health Metrics and Evaluation (IHME), an independent global health research center at the University of Washington, estimated that by mid-May of 2021, total worldwide COVID-19-related deaths may be as high as 7.1 million [4].

COVID-19 patients display a wide range of mild to severe symptoms, including fever, chills, dry cough, shortness of breath or breathing difficulty, fatigue, muscle aches, headache, loss of taste or smell, sore throat, nasal congestion, runny nose, conjunctivitis, nausea, vomiting, diarrhea, skin rash, and discoloration of fingers and toes [5,6,7]. These symptoms may appear within 2-14 days after exposure to the virus [6]. For most people, the symptoms are mild-to-moderate, but severe illness and death may occur in some, including older adults and patients with underlying medical conditions, such as obesity, kidney disease, heart disease, type-2 diabetes, cancer, COPD, sickle cell disease, or if they are immunocompromised [8]. In the US, COVID-19 deaths in young adults (age 25–44) have also been reported [9]. In early November 2020, the CDC also identified pregnant women and children with chronic kidney disease or sickle cell disease among the high-risk category as they can also develop a severe illness due to COVID infection. Since this is a new disease, scientists and healthcare workers are still on the learning curve but understanding the disease more each day.

From the very beginning of the coronavirus pandemic, scientists and health care workers were baffled about why some SARS-CoV-2 positive patients present mild-to-severe symptoms of COVID-19 infection, yet a significant segment of the infected population remains asymptomatic. These silent spreaders can effectively transmit the virus and thus make the epidemic further difficult to control [10]. Studies indicate that about 40% of the COVID-19 population remains asymptomatic [11,12]. Long et al. (2020) reported 37 asymptomatic SARS-CoV-2 positive cases in Wenzhou, China. These individuals did not have relevant clinical symptoms during their hospitalization period, and the duration of viral shedding ranged between 15 and 26 days [10]. The authors also reported that the asymptomatic individuals displayed reduced IgG and anti-inflammatory cytokine levels as well as a weaker immune response to SARS-CoV-2 infection. In the USA, studies revealed that in Arkansas, North Carolina, Ohio, and Virginia prisons, 96% of 3,277 inmates infected with COVID -19 were found to be asymptomatic [11]; in a homeless shelter in the Boston area, 88% of 147 COVID-19-infected residents were asymptomatic; similarly, 95% of 481 COVID-19 infected workers at a Tyson Foods plant were found to be asymptomatic [12].

Furthermore, COVID-19 has disproportionately affected some countries more than others. For example, in the USA, UK, and Italy, the infection and fatality rates are much higher as compared to Japan, Singapore, Malaysia, Indonesia, Thailand, and many other countries [13]. Understanding and unraveling these mysteries might be greatly significant as the answers may lead to the development of more effective therapeutics and vaccines in treating this deadly disease. This paper will focus on what could be the explanation behind a large segment of the SARS-CoV-2 positive population not showing manifestations of the disease and how to comprehend the disproportionate infection and mortality rates among various world populations.

## 2. Probable Explanations for Why SARS-CoV-2 Positive Individuals Remain Asymptomatic or Have Mild Symptoms

### 2.1. COVID-19 Immunity from Other Unrelated Vaccinations

As of this writing, SARS-CoV-2 has infected 221 countries in the world. However, some puzzling differences have been observed in morbidity and mortality, depending on the location. For instance, Miller et al. (2020) has reported that despite restrictive social isolation measures in Italy as compared to Japan, COVID-19 mortality was lower in Japan [14]. The authors suggested that the country-by-country variation in COVID-19 morbidity and mortality can be partially linked to the Bacillus Calmette-Guérin (BCG) vaccination policy. BCG is a live attenuated form of *Mycobacterium bovis* used as a vaccine for tuberculosis (TB) across the world. In laboratory studies, the BCG vaccine has been shown to induce non-specific immune responses by boosting the production of gamma interferon (IFN-γ) from CD4^+^ T-lymphocyte cells in mice treated with vaccinia virus [15]. Many nations, including Japan and China, have universal BCG vaccination programs, but Italy and the USA have never adopted the same policy [14]. Some countries such as Argentina, Brazil, Mexico, Poland, and Romania have discontinued universal BCG vaccination [16]. Recent reports indicate that BCG treatments could have beneficial effects on multiple diseases, including those that are autoimmune in nature [17].

A recent study suggested that BCG vaccination could provide protection against COVID-19 [18]. The authors indicated a strong association between the BCG index (the degree of BCG vaccination in a country) and COVID-19 mortality and reported that for every 10% increase in the BCG index, there was a 10.4% reduction in COVID-19 mortality. A 2020 study proposed a hypothesis that even unrelated live attenuated vaccines, such as BCG and MMR (measles, mumps, and rubella) vaccines could provide nonspecific protection against lethal infections and thus could be used as a preventive measure against COVID-19 [19]. The authors advocated that clinical trials with BCG and MMR could be a preventive measure in saving the lives of COVID-19 patients. Several other studies also indicated that unrelated vaccines such as BCG could provide some protection against COVID-19 [20,21,22].

For the prevention of measles, mumps, and rubella, the Advisory Committee on Immunization Practices (ACIP) recommends two doses of MMR vaccine routinely for children between the ages of 12 and 15 months and between 4 and 6 years. The MMR vaccine induces an immune response and triggers the production of IgG antibodies [23]. A 2020 report suggested that there could be a correlation between the levels of response to the MMR vaccine and the severity of symptoms in COVID-19 patients [24]. Individuals with higher levels of an immune response to the MMR vaccine might be better protected from SARS-CoV-2 infection and thus will have less severe COVID-19 symptoms. The mechanism associated with this protection is far from clear. One group of researchers performed homology analyses between the spike and nucleocapsid proteins of SARS-CoV-2 and the viruses in the MMR vaccine. They found similarities between the receptor-binding protein of the surface glycoprotein of SARS-CoV-2 and the measles fusion glycoprotein [25]. However, in another study involving 50 COVID-19 patients under the age of 42 who received the MMR vaccine as children, it was revealed that those having the highest antibody titers to the mumps virus were asymptomatic to COVID-19 infection [26]. In that study, there was no protection via anti-rubella or anti-measles virus antibody. Thus, the means by which the MMR vaccine provides protection has not been elucidated.

Root-Bernstein (2020) reported pneumococcal vaccines provided protection against infection and death to COVID-19 [27]. In this case, the effects were not due to polysaccharides but due to potential cross-reactivity between *Streptococcus pneumonaie* surface proteins and SARS-CoV-2 spike proteins, membrane protein, and replicase [27]. Another report noted that antibodies to the polio vaccine also provided protection against SARS CoV-2 infection [28]. In this case, cross-reacting antibodies against the RNA-dependent-RNA polymerase of poliovirus and SARS-CoV-2 were involved.

### 2.2. Memory T Cell-Mediated Immunity

Bert et al. (2020) reported that memory T cells have the potential to respond to SARS-CoV-2. They studied T cell responses against a nucleocapsid protein (N protein—a structural protein of SARS-CoV-2) in individuals recovering from COVID-19 and found that CD4^+^ and CD8^+^ T cells were reactive to the N protein. They also reported the presence of long-lasting memory T cells in individuals who recovered from the original SARS-CoV infection of 2003. These memory T cells showed strong responsiveness to SARS-CoV-2 N protein [29].

A recent report also indicated that the number and quality of SARS-CoV-2-specific memory T cells were factors in protection against the disease [30]. They also noted that asymptomatic individuals (and those without any detectable infection) had similar levels of memory T cells against the virus. This supports an earlier report that symptoms and recovery to COVID-19 were associated with stronger T-cell immunity [31].

### 2.3. ACE2 Receptors

**Figure 1:**
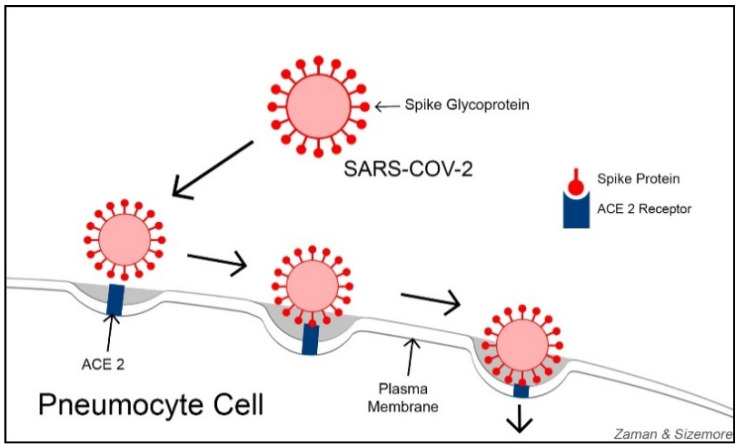
Spike glycoproteins of SARS-CoV-2 binding to ACE2 receptors on the plasma membrane before entering and infecting pneumocytes.

In the quest for developing an effective COVID-19 treatment, some researchers are focusing on ACE2 (Angiotensin-Converting Enzyme 2) receptors. With varying degrees, ACE2 receptors are expressed on the cells of nearly all human organs but are primarily found on alveolar epithelial cells, myocardial cells, proximal convolution tubular cells of kidney nephrons, bladder cells, and enterocyte cells in the ileum of the small intestine [32]. ACE2 receptors function as viral entry points, helping SARS-CoV-2 gain access to the cells. SARS-CoV-2 primarily damages the lung tissues and other organ tissues with abundant ACE2 receptors. Lung alveoli contain both Type-I and Type-II pneumocyte cells. Type-I pneumocytes (squamous epithelial cells for alveolar structures) constitute 95% of the alveolar wall, while Type-II pneumocytes (cuboidal glandular epithelial cells to produce surfactants) make up about 5% of the alveolar wall. ACE2 is highly abundant on Type-II pneumocytes. Using their spike proteins, SARS-CoV-2 viruses bind to ACE2 receptors before entering and infecting the cells (Figure 1). Some researchers consider understanding the functional relationship between ACE2 receptors and coronaviruses as key to developing effective treatments against COVID-19 [33].

**Figure 2:**
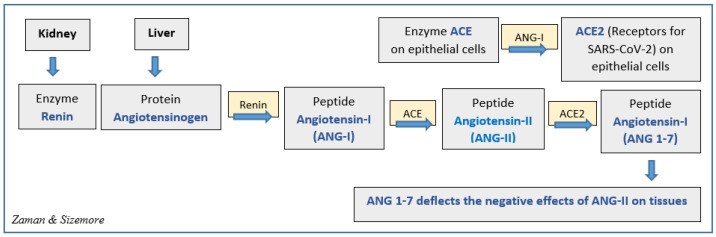
Possible role of ACE2 receptors and angiotensin peptide hormones in SARS-CoV-2 infection.

Although it is recognized that ACE2 receptors are involved in SARS-CoV-2 infection of epithelial cells of the lungs and other organs, the relationship between the quantity of the ACE2 receptors and the infectivity of SARS-CoV-2 has yet to be determined. Research indicated [8] that patients with chronic diseases were more vulnerable to COVID-19, and some studies suggested that patients with heart diseases, diabetes, and hypertension might have a higher number of ACE2 receptors [33]. However, there is no evidence that this is the primary reason patients with chronic diseases are more susceptible to SARS-CoV-2. Similarly, this is not counter evidence that individuals with a lower number of ACE2 receptors are less responsive to SARS-CoV-2. Studies with mice revealed that the lack of ACE2 was linked to non-viral-related lung, heart, or other tissue injuries [34]. On the contrary, studies indicated that by converting angiotensin-II into angiotensin-I (Figure 2), ACE2 could deflect the harmful actions of angiotensin II (ANG II), such as vasoconstriction, oxidative stress, fibrosis, hypertrophy, and inflammation of the tissues; therefore, it could provide protection against tissue and organ damages [32,33].

Perhaps, the abundance of ACE2 on cell membranes provides two-fold damage to the cells from SARS-CoV-2 infection: (1) abundant ACE2 receptors on epithelial cells provide the virus with numerous entry points to the cells, and (2) since the virus occupies most of the ACE2 receptors, the remaining number is inadequate to convert enough ANG II into ANG I to deflect the tissue damages by ANG II. Thus, both mechanisms might be responsible for the death of infected cells, causing the destruction of tissues and leading to organ failure. In a study published in 2020, Sriram and Insel argued that the level of ANG II activity determines the severity of damage in COVID-19 patients [35,36]. Angiotensin 1-7 (Ang 1-7) is an active heptapeptide (H-Asp-Arg-Val-Tyr-Ile-His-Pro-OH). It is produced from the polypeptide Angiotensin 1 by the action of the enzyme neprilysin or from hydrolysis of Ang II by ACE2 action [37]. Ang 1-7 is known to be a vasodilator with antioxidant and anti-inflammatory effects [38,39] and counteracts the actions of Ang II [40].

SARS-CoV-2 also employs the cell surface transmembrane protease serine 2 (TMPRSS2) in order to prime the spike protein, which is necessary for binding to ACE2 [41,42,43,44]. TMPRSS2 is expressed on several host cells, including nasal, bronchial, and testes tissues [42,43]. Furthermore, the prostate expressed very high levels of TMPRSS2, and one study showed that androgens regulated the expression of both TMPRSS2 and ACE2 [43,44]. It was thought that since older men tended to have more severe symptoms of COVID-19, it was plausible that increased TMPRSS2 expression was involved in the disparity [43]. However, Baughn et al. [44] showed that the expression of ACE2 and TMPRSS2 on several tissues was similar in both men and women. They suggested that although the differences in expression of these markers may not explain COVID-19 severity between genders, therapy targeting the activity of TMPRSS2 could be used as an alternate treatment [44].

### 2.4. Deficiency in Type I and Type III Interferons

Several articles have implicated a deficiency in Type I interferons (IFN-α and IFN-β) and the release of proinflammatory cytokines as factors in more severe forms of the disease [45,46]. However, the possible reasons for the lack of Type I IFN responses may involve several widely different mechanisms. The antiviral activity of the Type I interferons has long been noted as being critical in any viral response. However, SARS-CoV-2 can evade and counteract this activity by inhibiting both the release and antiviral activity of interferons [46]. Others have reported that individuals with inherited mutations in genes that regulate IFNs were more prone to life-threatening disease [47]. Bastard et al. (2020) noted the production of autoantibodies to Type I IFNs in SARS-CoV-2 infected patients, which appear to neutralize the activity of the cytokines [48]. Regardless of the reason for decreased Type I interferons, the lack of these cytokines results in increased viral replication followed by inflammation and a “cytokine storm” of proinflammatory cytokines [48]. This leads to an accumulation of monocytes that creates lung pathology, tissue damage, and decreased T cell responses [46]. A few studies have attempted clinical trials using Type I IFN in COVID-19 patients where one reported an increased discharge rate and decreased mortality in patients [49], while another [50] noted that treatment using IFN-α2b with or without arbidol reduced the presence of virus in the upper respiratory tract and decreased levels of proinflammatory IL-6 and C-reactive protein (CRP).

The Type III interferons (IFN-γ group) are preferentially expressed in the epithelial cells of the respiratory and gastrointestinal tract and promote antiviral activity without many of the proinflammatory effects of the Type I interferons [51]. As noted previously, SARS-CoV-2 can block IFN production, and COVID-19 patients have also shown decreased levels of Type III interferons [52,53]. Interestingly, one report noted that morbidity of COVID-19 was associated with increased expression of Type I and Type III interferons in the lungs [54]. Clinical trials using Type III interferons suggest that it may be harmful if given later in the disease [54]. It was also noted that Type III interferon may alter the protective barrier of the lung, causing the formation of bacterial superinfections [55]. This and the high toxicity often associated with IFN-γ therapy has led to caution in regards to Type III interferon therapy [52,55,56].

### 2.5. Cross-Reacting Antibodies to Other Coronaviruses

Some studies have suggested that partial protection against COVID-19 could be obtained from prior exposure to other coronaviruses that cause seasonal colds [57]. In one study, immunoglobin G (IgG) antibodies that could cross-react with the S2 subunit of the SARS-CoV-2 spike protein were found in some SARS-CoV-2 uninfected subjects. These antibodies had the ability to neutralize SARS-CoV-2. It was further noted that children and adolescents, who are more prone to common colds, such as those that are seasonal human coronavirus infections, had more of these types of antibodies. The authors noted that while a recent cold could provide some immunity, the antibody titer might not be protective against COVID-19. A second study also noted that antibodies to non-COVID-19 coronaviruses cross-reacted with SARS-CoV-2 [58]. They reported that samples collected from donors from sub-Saharan Africa were found to have more of the cross-reacting antibody than those from the USA. The authors noted that this was due to human coronaviruses being more prevalent in sub-Saharan Africa than in the United States and may explain why infections and deaths due to COVID-19 were comparatively low. It has also been noted that cross-reacting antibodies to human cold coronaviruses could result in false positive COVID-19 testing [59]. Thus, it is feasible that many COVID-19 tests resulted in faulty diagnoses due to previous exposure to the common cold.

### 2.6. Differences Due to Age

Numerous studies have pointed to differences in the age of patients as a key factor in the response to SARS-CoV-2. Cases of COVID-19 were seven times lower in younger children [26]. In general, adults were more prone to severe ARDS (acute respiratory distress syndrome), whereas children normally had milder symptoms, although some had multisymptom inflammatory syndrome (MIS-C) [60]. As with adults, not all children with COVID-19 have the same manifestations. One possible reason for children having fewer symptoms is that they have lower numbers of ACE2 receptors in their nasal epithelium [61]. Thus, some children may have more or less entry points for the virus than other children, and this may be associated with why some develop the disease. Whether or not children with COVID-19 were obese or had other health issues was not noted [60].

It is well known that antibody levels wane over time and that more recent immunization will result in higher titers of antibody. Children normally have had the MMR vaccine more recently and, thus, have more anti-mumps viral antibody [26]. Furthermore, if the polio vaccine also provides protection, then those who received the vaccine and are younger would have more available antibody that is cross-reactive. Furthermore, as previously noted, children and younger adults generally have more seasonal human coronavirus infections and thus have more cross-reacting antibodies [57].

## 3. Discussion and Conclusions

Patients with COVID-19 have presented a wide range of symptoms and sequela [4,5,6]. In general, those who were older and/or had underlying medical conditions were more prone to serious and, in many cases, life-threatening illnesses [20]. It is clear that having a strong Type I interferon response was a key factor in those who had mild-to-absent responses to SARS-CoV-2 [45,46].

Besides decreased Type I IFN in older individuals, the disparity in age-related cases could be due to a multitude of other factors in young people, including fewer ACE2 receptors, less likelihood of cytokine storm [61], and the presence of cross-reacting antibodies due to infection with non-COVID coronaviruses that cause the common cold [57]. In addition, cross-reacting antibodies to the mumps virus (via the MMR vaccine), the poliovirus [28], or pneumococcal bacteria (Root-Bernstein, 2020) [27] could also provide some protection, although it is not clear if this would fully protect someone against having COVID-19 [26]. Since the polio vaccine and the MMR vaccine are normally given to younger children, it is feasible that they would have more available cross-reacting antibodies than adults, for whom the antibody titers would have waned [26,62]. In addition to children younger than two years of age, the pneumococcal vaccine is recommended for the elderly [63]. Lewnard et al. (2021) reported reduced incidence of COVID-19 in adults aged 65 or older who had received the PCV13 vaccine, which is normally given to those who are two years old, whereas the PPSV23 vaccine (normally given to older individuals) did not provide protection [64]. The presence of various cross-reacting antibodies has also resulted in false-positive diagnoses of COVID-19 [59]. As of this writing, there are several SARS-CoV-2 variants of concern. Health officials and researchers are currently studying the effects of these variants, their susceptibility to vaccines, and whether they present new or different manifestations. At present, the evidence is preliminary, although it is developing rapidly [65].

Because of previous and widespread vaccination, we no longer immunize anyone against smallpox, which was declared eliminated in 1980 [66]. The polio virus was eliminated in the western part of the world in 1994 but remains endemic in parts of Afghanistan, Nigeria, and Pakistan [67]. As noted previously, vaccination against polio still continues today in the United States. It is hoped that with effective vaccination policies for COVID-19, the world will develop herd immunity and be protected against future pandemics. However, this will take considerable cooperation on the part of individuals and countries in receiving and administering the protective vaccines against SARS-CoV-2.

## Data Availability

Not applicable.
